# The role of M2 proteins of pneumoviruses in transcription regulation, prevention of hypermutation, and activation of the type I interferon pathway

**DOI:** 10.1128/jvi.01243-24

**Published:** 2025-01-21

**Authors:** Pau Ribó-Molina, Kevin Groen, Balasubramanian Susma, Stefan van Nieuwkoop, Mathis Funk, Ron A. M. Fouchier, Bernadette G. van den Hoogen

**Affiliations:** 1 Department of Viroscience, Erasmus Medical Center 686104 , Rotterdam, the Netherlands; University Medical Center Freiburg, Freiburg, Germany

**Keywords:** human metapneumovirus, M2 proteins, M2-2 protein, transcription, interferon antagonist

## Abstract

Human metapneumovirus (HMPV) is an important causative agent of respiratory tract disease. Fundamental knowledge of the interaction between HMPV and the innate immune system could lead to the design of novel antiviral therapies. Previously, we demonstrated that HMPV M2-2 deletion mutants had hypermutated genomes and contained defective interfering particles (DIs), which are potent inducers of the IFN response. Here, we investigated the role of the HMPV M2-2 protein as IFN antagonist using chimeric HMPV expressing M2 proteins of other pneumoviruses: respiratory syncytial virus (RSV) and avian metapneumovirus type C (AMPV/C). Chimeric HMPVs expressing the M2 proteins of RSV or AMPV/C were attenuated in HEp-2 cells but did not activate the IFN response, and their genomes were not hypermutated. In contrast, chimeric HMPVs expressing the M2-2 proteins of RSV and AMPV/C, in combination with HMPV M2-1, did activate the IFN response, and their genomes were hypermutated. Investigation of the role of the pneumovirus M2 proteins in transcription regulation demonstrated that the M2-2 protein, only in concerted action with autologous M2-1 protein, acted as a transcription elongation factor. As a second approach, chimeric RSV in which the IFN antagonists NS1 and NS2 were replaced by the HMPV M2-2 gene failed to suppress an IFN response, indicating that the HMPV M2-2 protein is not a potent IFN antagonist. These data indicate that expression of autologous M2-1 and M2-2 proteins is important for the fidelity of the RNA-dependent RNA polymerase, necessary to prevent the accumulation of mutations, and possibly DIs, thereby preventing activation of the IFN responses.

**IMPORTANCE:**

The M2-2 protein of human metapneumovirus is suggested to function as a type I interferon antagonist, a function so far not assigned to the M2 proteins of other pneumoviruses. Although M2-2 deletion mutants of HMPV activate the type I interferon pathway, these mutants have hypermutated genomes and contain defective interfering RNAs, known to activate the interferon pathway. Here, we show that the M2-2 protein, in concerted action with autologous M2-1 protein, acts as a transcription elongation factor, which could explain the accumulation of DIs in M2-2 deletion mutants. Additionally, chimeric RSV in which the IFN antagonists NS1 and NS2 were replaced by the HMPV M2-2 gene failed to suppress an IFN response. These data indicate that expression of autologous M2-1 and M2-2 proteins is required for the fidelity of the RNA-dependent RNA polymerase to prevent genome hypermutation and activation of the type I IFN pathway.

## INTRODUCTION

Human metapneumovirus (HMPV) is a negative-sense single-stranded RNA (-ssRNA) virus, belonging to the *Pneumoviridae* family, that causes respiratory tract infections mainly in the elderly, immunocompromised individuals and infants (reviewed in [1]). Currently, no vaccines or antiviral treatments are available. Increased fundamental knowledge of HMPV could lead to the design of novel antiviral therapies.

The response of mammalian cells to RNA virus infection is characterized by the production of type-I interferons (IFNs) (2).To overcome this cellular antiviral response, most—if not all—mammalian viruses have mechanisms to avoid induction of IFN production (3). For example, the influenza A virus NS1 protein and the respiratory syncytial virus (RSV) NS1 and NS2 proteins have been described as IFN antagonists (4–6). For HMPV, the attachment glycoprotein (G) and small hydrophobic (SH) protein have been proposed as IFN antagonists (7–10). However, we recently demonstrated that these two proteins do not qualify as bona fide IFN antagonists (11). The M2-2 protein has also been proposed as an IFN antagonist (12–15). Although it has indeed been shown that M2-2 deletion mutant viruses induce IFN production (11, 14), we, and others, have shown that the genomes of M2-2 deletion mutant viruses contained hypermutated regions (11, 16) and that M2-2 deletion mutants accumulated defective interfering particles (DIs) during the generation of virus stocks (11). Given that DIs are potent inducers of the IFN response, the role of the M2-2 protein as bona fide IFN antagonist remains elusive.

Here, we aimed to investigate the role of M2 proteins of Pneumoviruses in preventing the hypermutation of genomes, accumulation of DIs, and subsequent activation of the IFN response using chimeric HMPV expressing M2 and/or M2-2 proteins of HMPV, RSV, or avian metapneumovirus type-C (AMPV/C). We show that the role of M2 proteins as transcription regulatory factors is related to the prevention of accumulation of hypermutated genomes and DIs, and therefore activation of the IFN response. In addition, we show that the HMPV M2-2 protein does not function as a potent IFN antagonist when expressed by chimeric RSV in which the NS1 and NS2 protein genes were replaced by the HMPV M2-2 protein gene.

## RESULTS

### The RSV or AMPV/C M2 proteins can replace the function of the HMPV M2 proteins

**Fig 1:**
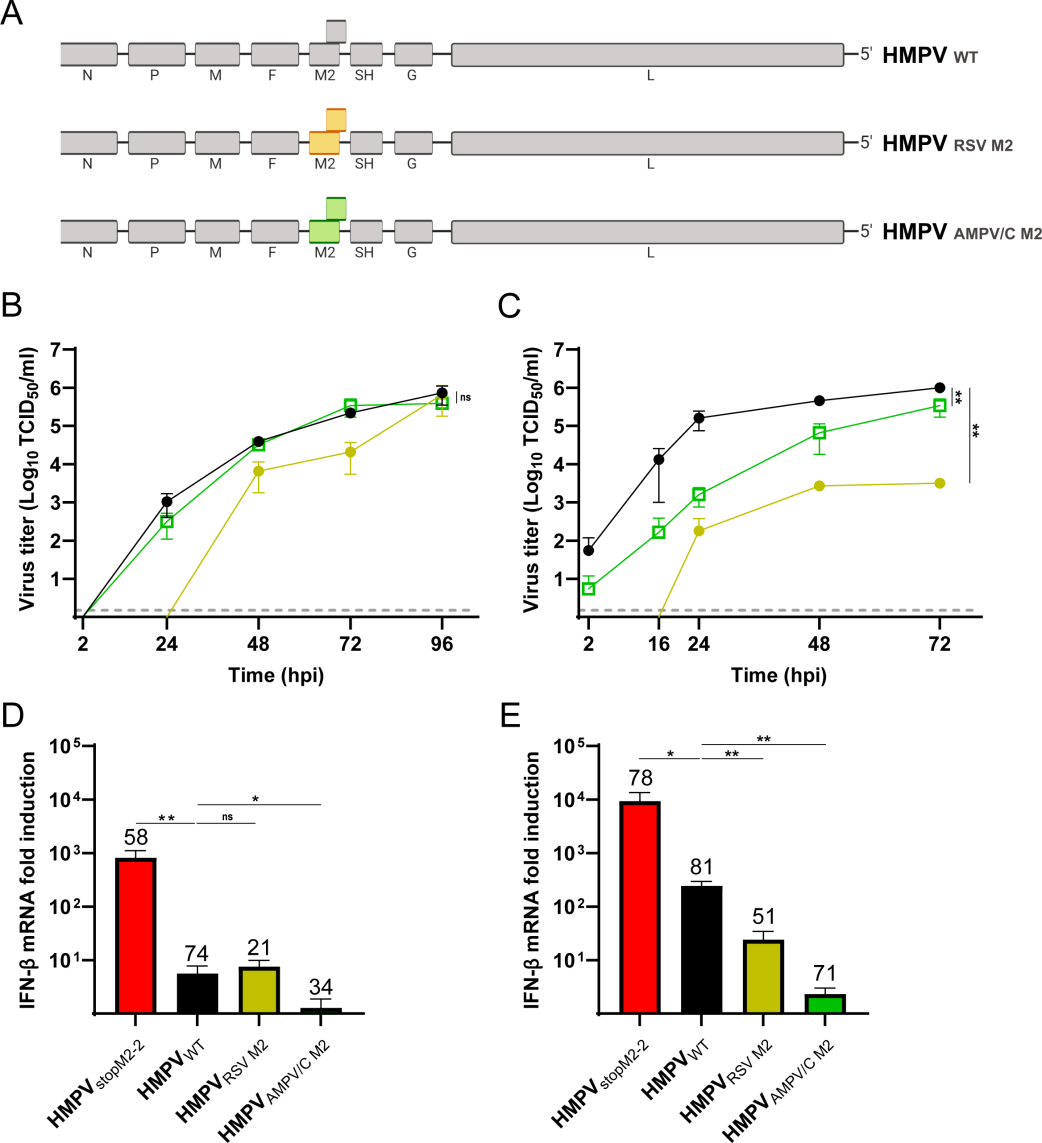
(**A**) Schematic representation of chimeric HMPV NL/1/00 viruses. The HMPV M2 gene was replaced by that of either RSV or AMPV/C with the gene-start (GS) and gene-end (GE) sequences of the HMPV M2 gene. (**B, C**) Replication kinetics of HMPV_WT_ (black circles), HMPV_RSV M2_ (yellow circles), and HMPV_AMPV/C M2_ (green open squares) in (**B**) Vero-118 cells or (**C**) HEp-2 cells. The limit of detection is shown with a gray dotted line at 1.5 TCID_50_/mL. (**D, E**) IFN-β mRNA expression levels at 24hpi in HEp-2 cells inoculated with HMPV_StopM2-2_, HMPV_WT_, HMPV_RSV M2_, or HMPV_AMPV/C M2_ at an MOI of (**D**) 3 or (**E**) 20. Numbers on top of the bars represent the percentage of infected cells. Image (**A**) was generated with Biorender. Figures B-E are representative of three individual experiments. Error bars indicate standard deviations. **=*P* < .01 and *=*P* < .05, as calculated by an unpaired *t* test.

To investigate whether the M2 proteins of AMPV/C or RSV are able to substitute the function of the M2 proteins of HMPV, chimeric HMPVs were generated with the complete M2 open reading frames (ORF) exchanged for those of RSV or AMPV/C (HMPV_RSV M2_ and HMPV_AMPV/C M2_, respectively) (Fig. 1A). In IFN-deficient Vero-118 cells, HMPV_AMPV/C M2_ replicate to similar levels as HMPV_WT_, and although HMPV_RSV M2_ had slower replication kinetics than HMPV_WT_, similar endpoint titers were reached (Fig. 1B). In IFN-competent HEp-2 cells, both chimeras were significantly attenuated compared with HMPV_WT_, although HMPV_AMPV/C M2_ replicated to higher titers than HMPV_RSV M2_ (Fig. 1C). We next assessed whether the M2 chimeric viruses activated the IFN response in HEp-2 cells. HMPV_StopM2-2_, not expressing M2-2 and previously shown to induce high levels of IFN-β expression in A549 cells (11), was used as a positive control. Indeed, upon inoculation of HEp-2 cells at a multiplicity of infection (MOI) of 3, HMPV_StopM2-2_ induced significantly higher IFN-β mRNA levels than HMPV_WT_ at 24 h post-inoculation (hpi). In contrast, both HMPV_RSV M2_ and HMPV_AMPV/C M2_ induced similar, or even significantly lower levels, of IFN-β mRNA than HMPV_WT_ (Fig. 1D). However, the proportions of infected cells for the chimeric viruses were relatively low (21% and 34% for HMPV_RSV M2_ and HMPV_AMPV/C M2_, respectively), and therefore, the experiment was repeated at a higher MOI. Inoculation of HEp-2 cells at an MOI of 20 resulted in a higher proportion of infected cells for all viruses compared with inoculation at an MOI of 3, at 24hpi (Fig. 1E). Upon inoculation at an MOI of 20, HMPV_WT_-induced higher IFN-β mRNA expression levels than upon inoculation at an MOI of 3, but still significantly lower levels than HMPV_StopM2-2_. The higher proportions of infected cells for the chimeric viruses (51% and 71% for HMPV_RSV M2_ and HMPV_AMPV/C M2_, respectively) did not result in a substantial increase in IFN-β mRNA expression levels, as these were even significantly lower than, in HMPV_WT_ inoculated cells (Fig. 1E). Therefore, the attenuated replication of the chimeric viruses in HEp-2 cells was not related to activation of the IFN response.

**Fig 2:**
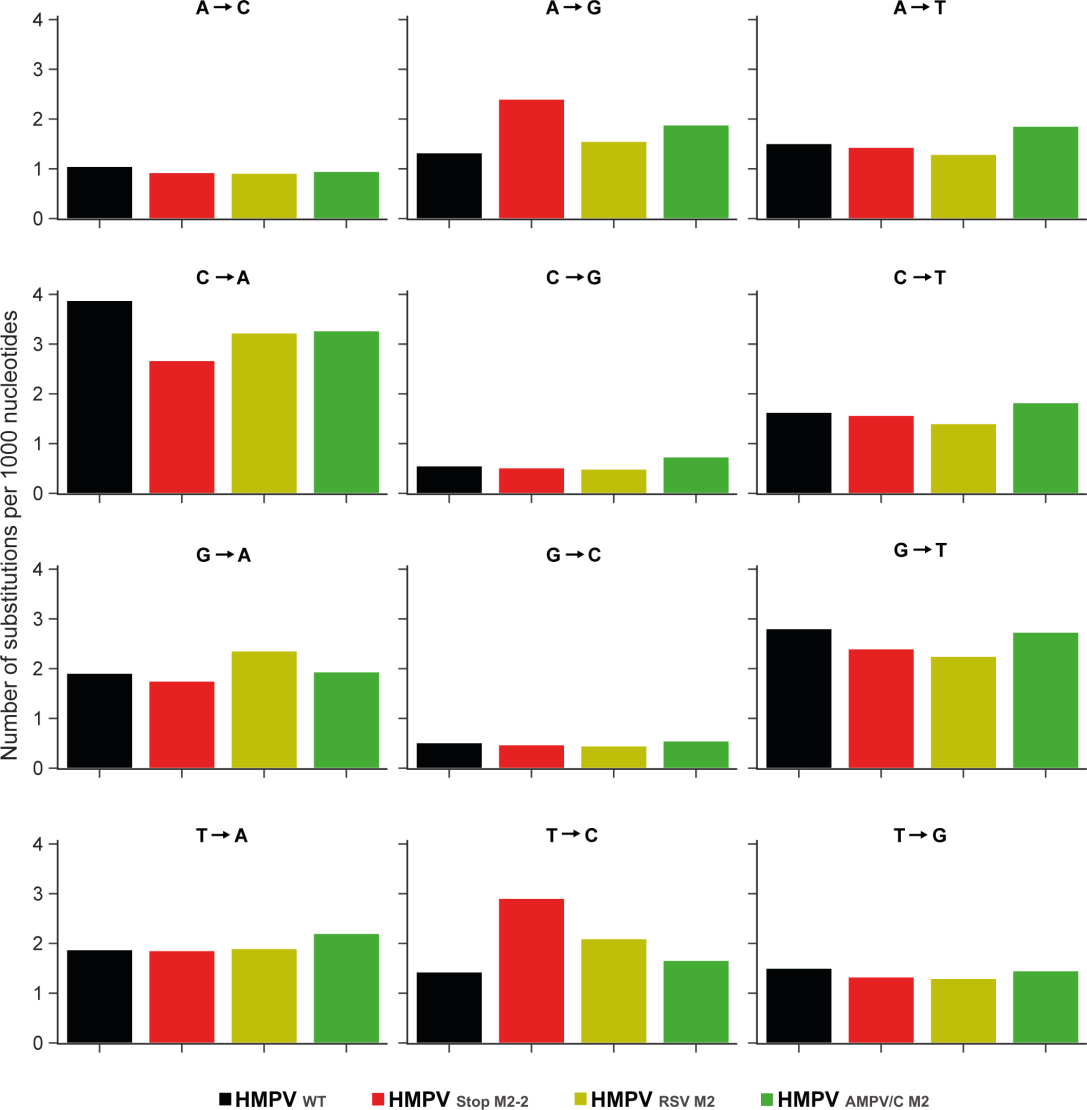
Analysis of Illumina next-generation sequencing of HMPV_WT_, HMPV_StopM2-2_, HMPV_RSV M2_, and HMPV_AMPV/C M2_ genomes. Reads were aligned to the reference using the CLC genomic workbench software and the number of nucleotide substitutions were quantified. Bars represent the specified number of substitutions per 1,000 nucleotides in HMPV_WT_, HMPV_StopM2-2_, HMPV_RSV M2_, and HMPV_AMPV/C M2_.

Previously, we demonstrated that the genomes of M2-2 deletion mutants and HMPV_StopM2-2_ were hypermutated. The majority of these mutations were A-to-G and T-to-C due to ADAR1 editing (11). To investigate whether the M2 proteins of RSV and AMPV/C were able to substitute the function of the M2 proteins of HMPV in preventing this hypermutation, virus stocks were subjected to next-generation sequencing. Analysis of the sequencing reads demonstrated that the genomes of HMPV_WT_, HMPV_RSV M2_, and HMPV_AMPV/C M2_ contained a low number of A-to-G and T-to-C nucleotide substitutions, in contrast to higher A-to-G and T-to-C substitution rates in HMPV_StopM2-2_ (Fig. 2). These results indicated that the M2 proteins of HMPV, RSV, and AMPV/C have a similar function in preventing hypermutation of the genome and activation of the IFN response. Interestingly, a higher C-to-A mutation rate was observed for HMPV_WT_ compared with HMPV_StopM2-2_ and M2 chimeric viruses, for which we have no explanation.

### Exchange of the HMPV M2-2 protein for that of RSV or AMPV/C results in activation of the IFN response

**Fig 3:**
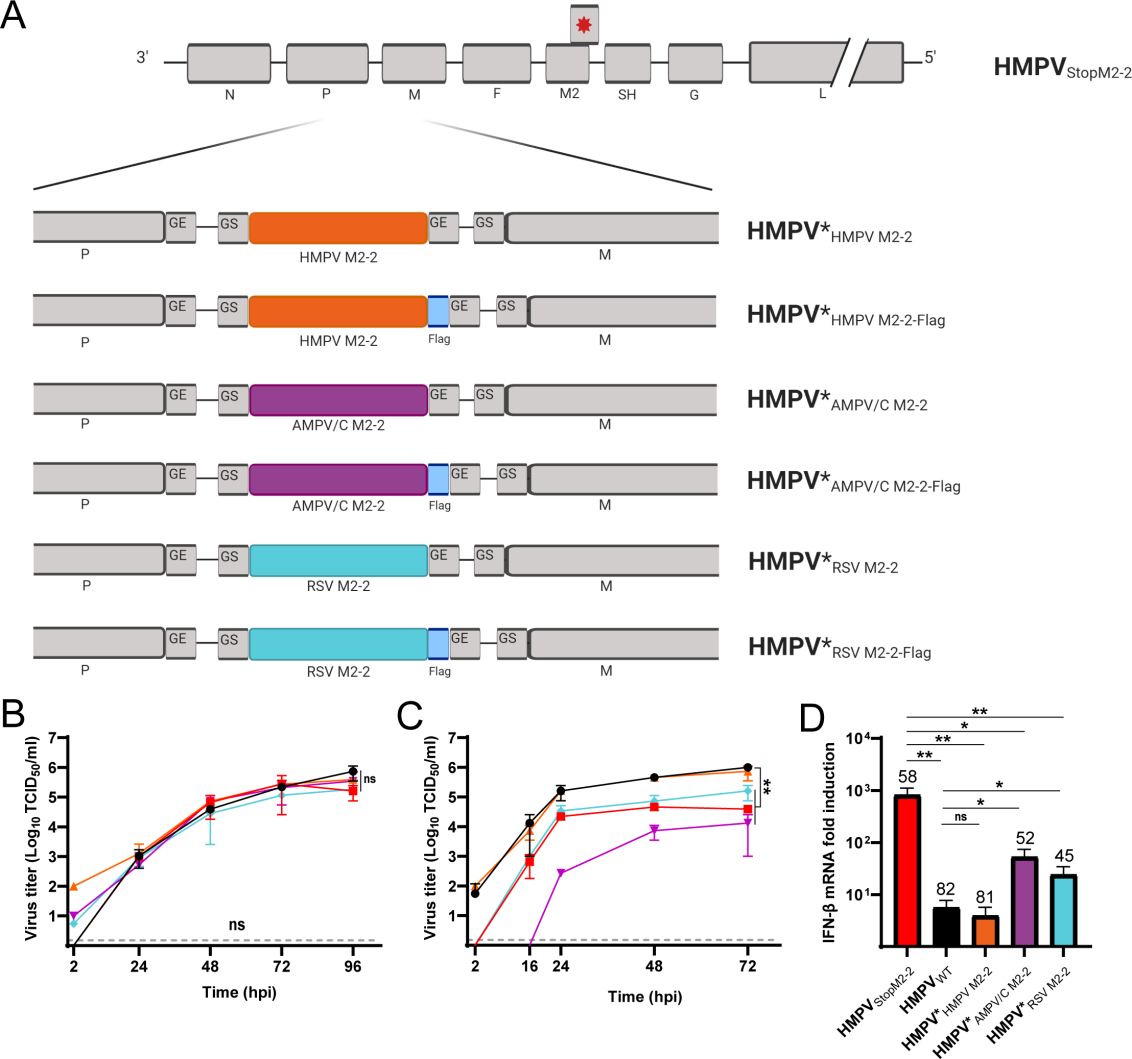
(**A**) Schematic representation of chimeric HMPV NL/1/00 viruses. To generate chimeric viruses expressing the M2-2 protein of RSV or APV-C, a gene cassette encoding either the HMPV, RSV, or AMPV/C M2-2 gene (with the gene-start and gene-end sequences of the HMPV *P* gene, either with or without a C-terminal flag tag) was cloned in between the *P* and M genes of the HMPV_stopM2-2_ cDNA clone. (**B, C**) Replication kinetics of HMPV_StopM2-2_ (squares), HMPV_WT_ (circles), HMPV*_HMPV M2-2_ (triangles up), HMPV_APV-C M2-2_ (triangles down), and HMPV_RSV M2-2_ (diamonds) upon inoculation of (**B**) Vero-118 cells or (**C**) HEp-2 cells. The limit of detection is shown with a gray-dotted line at 1.5 TCID_50_/mL. (**D**) IFN-β mRNA expression levels at 24 hpi upon inoculation of HEp-2 cells with HMPV_StopM2-2_, HMPV_WT_, HMPV*_HMPV M2-2_, HMPV_APV-C M2-2_, or HMPV_RSV M2-2_. Numbers on top of the bars represent the percentage of infected cells. Image (**A**) was generated with Biorender. Figures B-D are representative of three individual experiments. Error bars indicate standard deviations. **=*P* < .01 and *=*P* < .05, as calculated by an unpaired t test.

To address the function of the M2-2 protein separately from that of the autologous M2-1 protein, chimeric HMPVs were generated that expressed HMPV M2-1 but the M2-2 proteins of RSV or AMPV/C. As the pneumovirus M2-2 protein is expressed as the second ORF of the M2 gene, which partially overlaps with the M2-1 ORF, the exchange of the M2-2 ORFs at the original position was not possible without altering the M2-1 ORF. Therefore, chimeric HMPVs were generated in which the expression of the HMPV M2-2 protein at the original position was abolished (HMPV_StopM2-2_), and the genes for the M2-2 proteins of either HMPV, RSV, or AMPV/C were provided from the third position of the HMPV genome (HMPV*_HMPV M2-2_, HMPV*_RSV M2-2_, and HMPV*_AMPV/C M2-2_, Fig. 3A). Previously, it was shown that expression of foreign genes from this position did not change replication kinetics of the virus (17). Because antibodies against the HMPV M2-2 protein are not available, additional viruses were generated that expressed the M2-2 proteins with a fused C-terminal flag-tag sequence at the same position of the HMPV_StopM2-2_ genome (HMPV*_HMPV M2-2-flag_, HMPV*_RSV M2-2-flag_, and HMPV*_AMPV/C M2-2-flag_, Fig. 3A). These viruses were used to confirm expression of M2-2 proteins from this altered genome position (Fig. S1). In IFN-deficient Vero-118 cells, all mutant and chimeric viruses replicated to similar levels as HMPV_WT_ (Fig. 3B). In IFN-competent HEp-2 cells, replication of HMPV_StopM2-2_ was significantly attenuated compared with that of HMPV_WT_ (Fig. 3C), similar to previous observations in A549 cells (11). The identical replication kinetics of HMPV*_HMPV M2-2_ as HMPV_WT_ indicated that M2-2 protein expression from the third position in the HMPV_StopM2-2_ genome successfully rescued the wild-type phenotype. However, expression of either the RSV or AMPV/C M2-2 protein from the third position of the genome of HMPV_StopM2-2_ did not result in recovery of the wild-type phenotype, as both HMPV*_RSV M2-2_ and HMPV*_AMPV/C M2-2_ were significantly attenuated compared with HMPV_WT_ (Fig. 3C). Interestingly, the replication of HMPV*_AMPV/C M2-2_ was more profoundly attenuated than that of HMPV*_RSV M2-2_. Next, we assessed whether the chimeric M2-2 viruses activated the IFN response upon inoculation of HEp-2 cells, using HMPV_StopM2-2_ as a positive control. As expected, HMPV_StopM2-2_ induced significantly higher IFN-β mRNA expression levels than HMPV_WT_ at 24 hpi, despite a lower proportion of infected cells for HMPV_StopM2-2_ (58%) than for HMPV_WT_ (82%) (Fig. 3D). Inoculation with HMPV*_HMPV M2-2_ induced similar IFN-β mRNA expression levels as inoculation with HMPV_WT_, with similar proportion of infected cells (81%–82%), indicating that expression of HMPV M2-2 protein from the third position of the genome of HMPV_StopM2-2_ restored the wild-type phenotype (Fig. 3D). Inoculation with HMPV*_RSV M2-2_ or HMPV*_AMPV/C M2-2_ resulted in a lower proportion of infected cells (45%–52%), than inoculation with HMPV_WT_ (82%), but in significantly higher IFN-β mRNA expression levels than HMPV_WT_. Although inoculation of HEp-2 cells with the chimeric viruses resulted in a similar proportion of the infected cells as for HMPV_StopM2-2_ (58%), the chimeras induced approximately 10-fold lower IFN-β mRNA expression levels than HMPV_StopM2-2_, indicating that the expression of RSV and AMPV/C M2-2 protein from the third position in the genome of HMPV_StopM2-2_ only partially rescued the wild-type phenotype.

**Fig 4:**
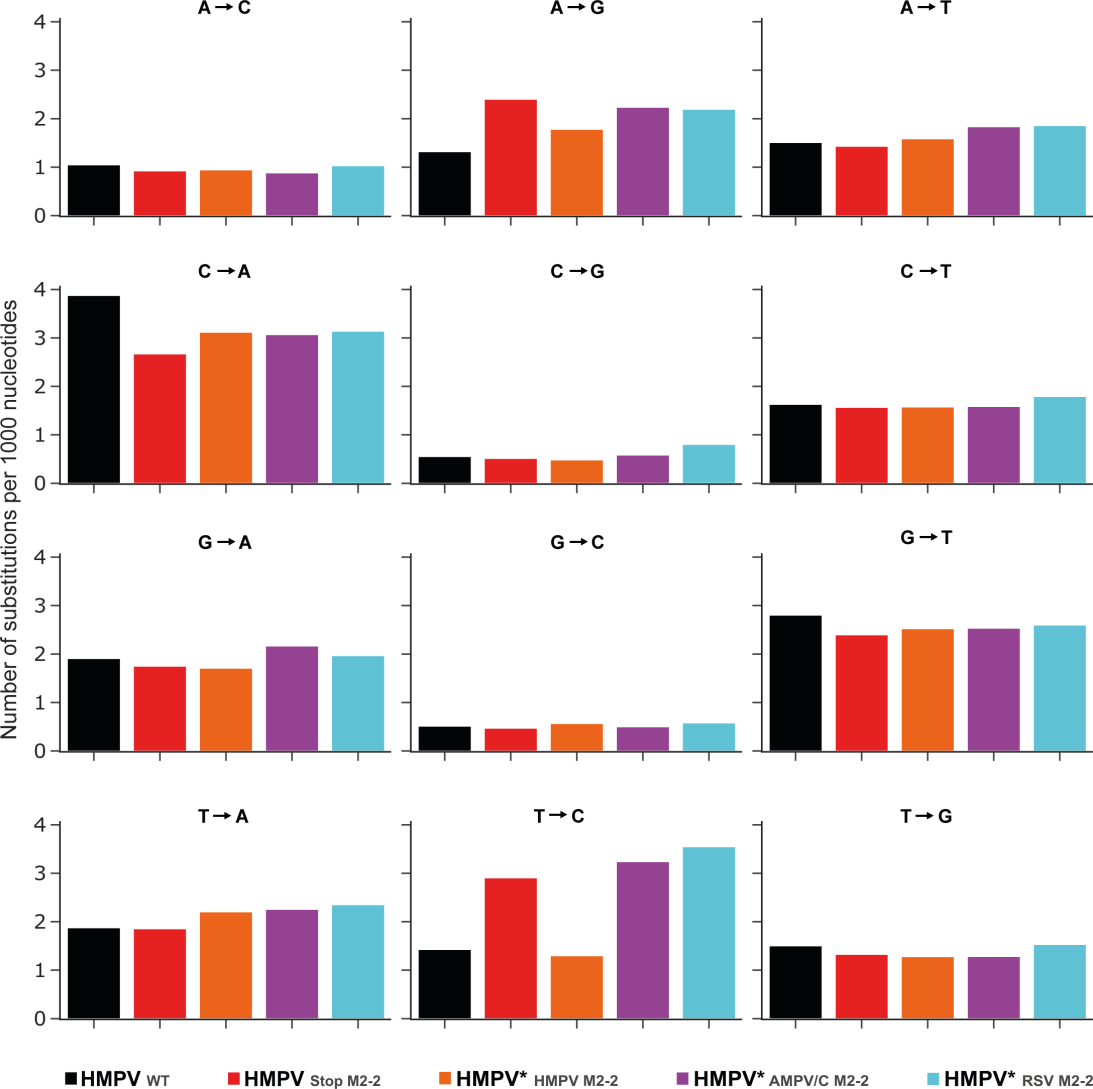
Analysis of Illumina next-generation sequencing of HMPV_WT_, HMPV_StopM2-2_, HMPV*_HMPV M2-2_, HMPV*_AMPV/C M2-2_, and HMPV*_RSV M2-2_ genomes. Reads were aligned to the reference using the CLC genomic workbench software, and the number of nucleotide substitutions was quantified. Bars represent the specified number of substitutions per 1,000 nucleotides in HMPV_WT_, HMPV_StopM2-2_, HMPV*_HMPV M2-2_, HMPV*_AMPV/C M2-2_, and HMPV*_RSV M2-2_.

To investigate whether the M2-2 chimeric virus genomes contained hypermutated genomes, the virus stocks were subjected to next-generation sequencing, and the mutation rates were assessed. Compared with the genomes of HMPV_WT_, the genomes HMPV_StopM2-2_ displayed a higher number of A-to-G and T-to-C nucleotide substitutions, as already shown in Figure 2 (Fig. 4). Expression of HMPV M2-2 from the third position of the genome (HMPV*_HMPV M2-2_) resulted in a similar number of A-to-G and T-to-C nucleotide substitutions in the genomes as observed in the HMPV_WT_ genomes, confirming that the position from which the M2-2 protein is expressed did not alter the virus phenotype. However, the genomes of both HMPV*_RSV M2-2_ and HMPV*_AMPV/C M2-2_ contained higher numbers of A-to-G and T-to-C nucleotide substitutions compared with the genomes of HMPV_WT_, indicating that complementation of the M2-2 proteins of RSV or AMPV/C from the third position of the genome did not rescue the wild-type phenotype.

### M2-2 chimeric HMPVs have altered transcription profiles

The replication of both HMPV_RSV M2_ and HMPV_AMPV/C M2_ was attenuated in HEp-2 cells, but both viruses did not activate the IFN response, indicating that attenuation of these viruses might be due to defects in transcription and replication. To investigate the role of the M2 proteins in the transcription process, the RNA expression levels of the HMPV nucleoprotein (N) and large polymerase (L) genes were determined by RT-qPCR at different time points after inoculation of HEp-2 cells with HMPV_WT_, HMPV_StopM2-2_, HMPV*_HMPV M2-2_, or the chimeric HMPVs. The C_T_ values were transformed into TCID_50_ equivalents using a standard curve derived from a titrated virus stock (Fig. S2), which were used to calculate the ratio of HMPV N and L transcripts. The N and L genes were chosen to compare RNA expression levels in relation to transcription processivity as they are the most extreme 3’ and 5’ proximal ORFs in the HMPV genome.

**Fig 5:**
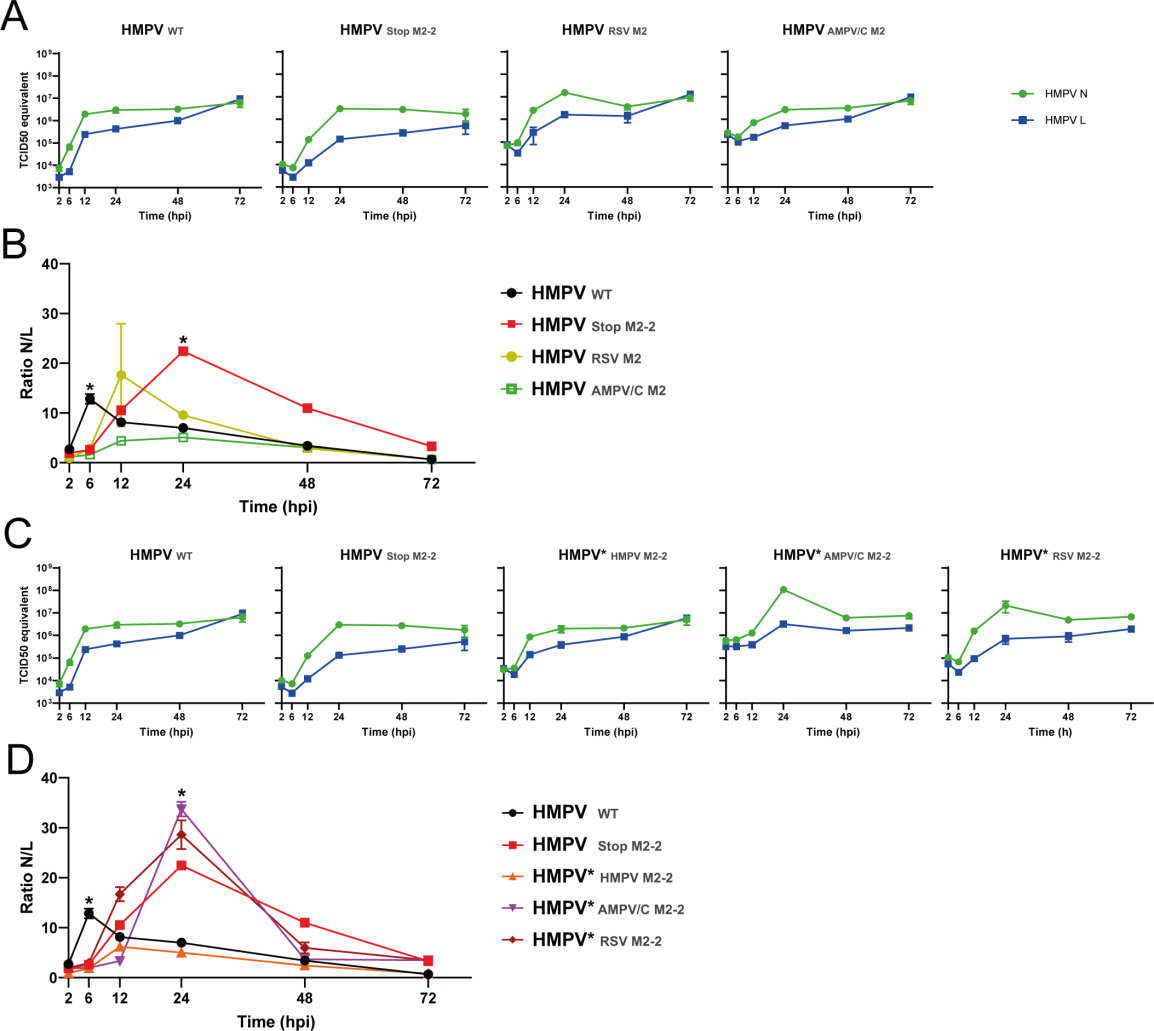
Expression levels of HMPV N and L RNA over time in HEp-2 cells. (**A-B**) Quantification of HMPV N and L TCID_50_ equivalent and ratio of N/L over time, during replication of HMPV_WT_, HMPV_StopM2-2_, HMPV_RSV M2_, and HMPV_AMPV/C M2_. (**C-D**) Quantification of HMPV N and L TCID_50_ equivalent and ratio of N/L over time, during replication of HMPV_WT_, HMPV_StopM2-2_, HMPV*_HMPV M2-2_, HMPV*_AMPV/C M2-2_, and HMPV*_RSV M2-2_. HEp-2 cells were inoculated at an MOI of 0.1, and RNA was isolated at the indicated hours post-inoculation (hpi). HMPV N and HMPV L RNA expression levels were determined by qRT-PCR, and C_T_ values were compared with a standard curve derived from a stock of HMPV NL/1/00 titrated on Vero-118 cells (see Fig. S2). ****=*P* < .0001, **=*P* < .01 and *=*P* < .05, as calculated by an unpaired t test.

For HMPV_WT_, the expression levels of N and L RNA increased from 2 to 12 hpi. Thereafter, the expression levels of N RNA remained higher than the expression levels of L until 72 hpi (Fig. 5A). At 6 hpi, the N/L expression ratio was significantly higher than those of the other viruses and decreased at later time points (12, 24, 48, and 72 hpi), indicating an increase in L RNA levels relative to N RNA levels at later time points (Fig. 5B). For HMPV_StopM2-2_, the N and L RNA expression levels increased up until 24 hpi, with a significantly higher N/L ratio than those of the other viruses at that time point (Fig. 5B). The N/L ratios for HMPV_StopM2-2_ decreased after 24 hpi but remained higher than those of HMPV_WT_ at 48 and 72 hpi. This indicated reduced transcription elongation from the N to L genes by viruses lacking M2-2 protein expression compared with that of HMPV_WT_. Although HMPV_StopM2-2_ displayed an altered transcription process compared with HMPV_WT_, the exchange of both HMPV M2 proteins with those of AMPV/C or RSV resulted in more similar transcription profiles as observed for HMPV_WT_ after 12 hpi (Fig. 5A and B), although the peak of the N/L ratio was 6 h later than that of HMPV_WT_. Expression of HMPV M2-2 from the third position of the genome in HMPV_StopM2-2_, HMPV*_HMPV M2-2_, restored the transcription profile to a similar profile as that of HMPV_WT_. Although the N/L ratio peak was lower and 6 h later than for HMPV_WT_, the profile after 12 h was similar (Fig. 5C and D). However, expression of either RSV M2-2 or AMPV/C M2-2 from the third position of the HMPV_StopM2-2_ genome did not restore the wild-type phenotype, as both chimeric viruses had an increase in N expression levels at 24 hpi, similarly to HMPV_StopM2-2_. At 24 hpi, the N/L ratios for HMPV*_RSV M2-2_ and HMPV*_AMPV/C M2-2_ were similar to those of HMPV_Stop M2-2_, which were all significantly higher than those of HMPV_WT_ and HMPV*_HMPV M2-2_ (Fig. 5D). Together, these data indicate a role for the M2-2 protein in regulating transcription, which likely requires a concerted action with the autologous M2-1 protein.

### The HMPV M2-2 protein cannot replace the IFN antagonistic function of the RSV NS1 and NS2 proteins

**Fig 6:**
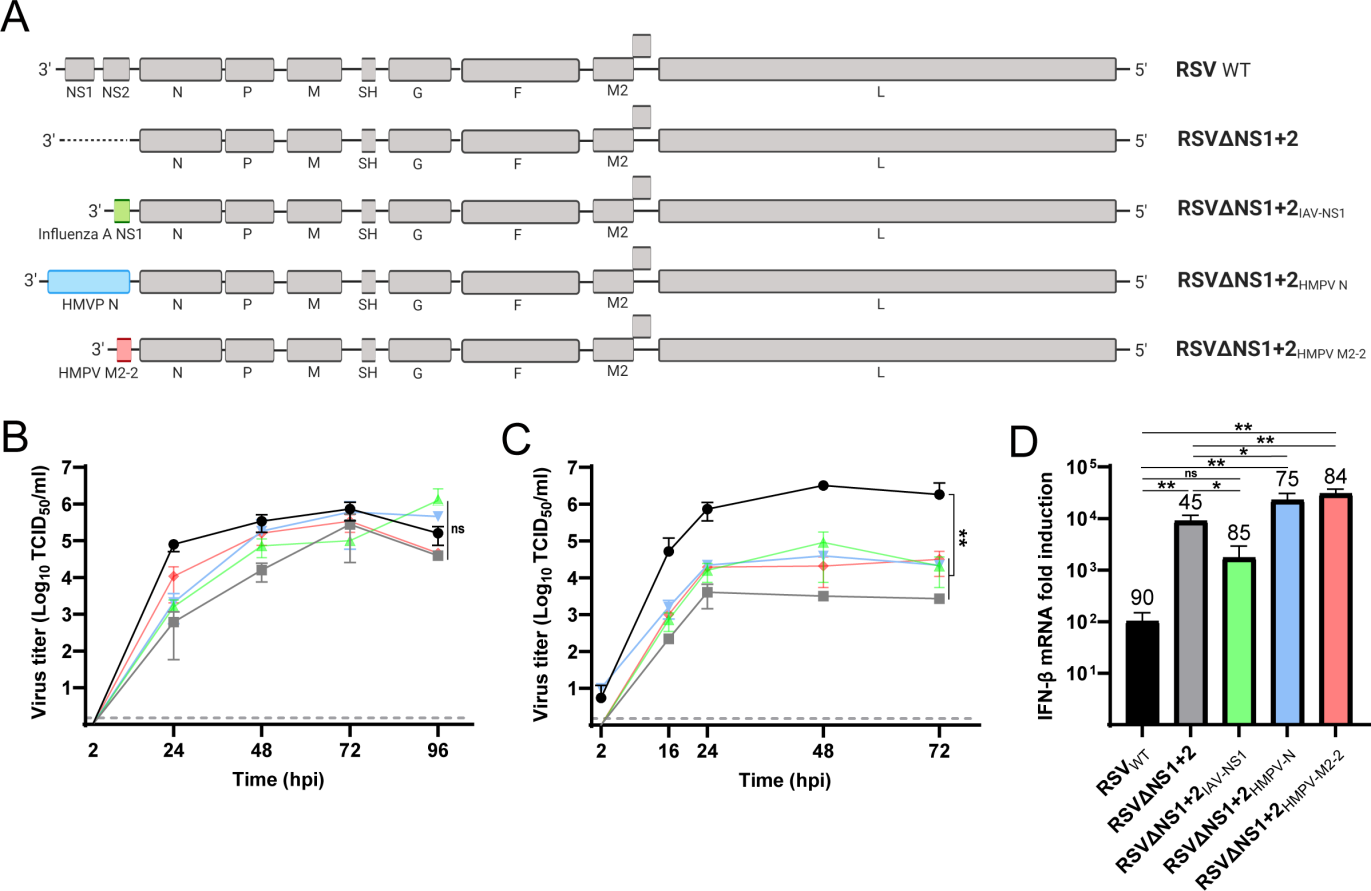
(**A**) Schematic representation of chimeric RSV viruses. Nonstructural genes NS1 and NS2 were either deleted or replaced by the Influenza A virus NS1, HMPV N, or HMPV M2-2 genes, combined with the gene-start and gene-end sequences of the RSV NS1 gene. (**B-C**) Replication kinetics of RSV_WT_ (circles), RSVΔNS1+2 (squares), RSVΔNS1+2_V NS1_ (triangles up), RSVΔNS1+2 _MPV N_ (triangles down), and RSVΔNS1+2_MPV M2-2_ (diamonds) in (**B**) Vero-118 and (**C**) HEp-2 cells. The limit of detection is shown with a gray-dotted line at 1.5 TCID_50_/mL. (**D**) IFN-β mRNA expression levels in HEp-2 cells inoculated with RSV_WT_, RSVΔNS1+2, RSVΔNS1+2_AV NS1_, RSVΔNS1+2_MPV N_, or RSVΔNS1+2_MPV M2-2_. Numbers on top of the bars represent the percentage of infected cells. Error bars indicate standard deviations. Image (**A**) was generated with Biorender. Figures B–D are representative of three individual experiments. **=*P* < .01, as calculated by an unpaired t test.

To investigate the role of the HMPV M2-2 protein as bona fide IFN antagonist in the absence of hypermutated genomes and DIs, chimeric RSVs were generated that expressed the HMPV M2-2 gene (RSVΔNS1+2_HMPV-M2-2_) or the influenza A virus (IAV) NS1 gene (RSVΔNS1+2_IAV-NS1_) instead of the RSV NS1 and NS2 genes, which are known to function as IFN antagonist (Fig. 6A). The NS1 protein of IAV was used as a positive control, as it is a potent bona fide IFN antagonist (4). Deletion of promoter-proximal genes from the RSV genome could alter the transcription process. To control for this, RSVΔNS1+2 expressing the HMPV N gene at the position of the NS1 and NS2 genes was generated (RSVΔNS1+2_HMPV-N_) as control. Protein expression was confirmed for the HMPV N protein (Fig. S3A through C), as well as for IAV NS1 protein (Fig. S3D), in the chimeric RSV. As reactive antibodies against the HMPV M2-2 protein are not available, the presence of M2-2 mRNA was detected to confirm the expression of HMPV M2-2 gene by RSVΔNS1+2_HMPV-M2-2_ (Fig. S3E).

Assessment of the replication kinetics in Vero-118 cells demonstrated slower, although not significant, replication for the chimeric viruses and RSVΔNS1+2 compared with RSV_WT_, but all viruses reached roughly similar end titers at 72–96 hpi (Fig. 6B). In IFN-competent HEp-2 cells, the replication of RSVΔNS1+2 was significantly attenuated compared with that of RSV_WT_ (Fig. 6C). However, in these cells, RSVΔNS1+2_IAV-NS1_, RSVΔNS1+2_HMPV-N_, and RSVΔNS1+2_HMPV-M2-2_ all had improved replication kinetics compared with RSVΔNS1+2 but still did not replicate to the same levels as RSV_WT_. The fact that RSVΔNS1+2_HMPV-N_ replicated to similar titers as RSVΔNS1+2_IAV-NS1_ might be related to a partial rescue of the transcriptional profile. Upon inoculation of HEp-2 cells, RSVΔNS1+2 induced significantly higher levels of IFN-β mRNA than RSV_WT_ at 16 hpi, despite the lower proportion of infected cells (45% and 90% for RSVΔNS1+2 and RSV_WT_, respectively) (Fig. 6D). In contrast, inoculation with RSVΔNS1+2_IAV-NS1_ resulted in higher proportions of infected cells than inoculation with RSVΔNS1+2 (85% for RSVΔNS1+2_IAV-NS1_ and 45% for RSVΔNS1+2), but RSVΔNS1+2_IAV-NS1_ induced lower IFN-β mRNA expression levels than RSVΔNS1+2. However, RSVΔNS1+2_IAV-NS1_ did induce slightly higher IFN-β mRNA expression levels, although not significantly, than RSV_WT_, indicating that the IAV NS1 protein only partially replaced the IFN-antagonistic functions of the RSV NS1 and NS2 proteins. Inoculation of HEp-2 cells with RSVΔNS1+2_HMPV-N_ and RSVΔNS1+2_HMPV-M2-2_ resulted in proportions of infected cells approaching those upon inoculation with RSV_WT_ (90% for RSV_WT_, 75% for RSVΔNS1+2_HMPV-N_, and 84% for RSVΔNS1+2_HMPV-M2-2_). However, RSVΔNS1+2_HMPV-N_ and RSVΔNS1+2_HMPV-M2-2_ induced significantly higher IFN-β mRNA expression levels than RSV_WT_ and similar levels as RSVΔNS1+2. Thus, the HMPV N and M2-2 proteins did not replace the function of the RSV NS1 and NS2 proteins as potent IFN antagonists.

## DISCUSSION

Although it has been suggested that the HMPV M2-2 protein acts as an IFN antagonist (12–15), it was recently demonstrated that M2-2 deletion mutant genomes were hypermutated and that these virus stocks contained DIs, which potently activate the IFN response (11). This finding complicates studies related to the interaction between HMPV M2-2 and the innate immune system. Here, we aimed to investigate the role of M2 proteins of pneumoviruses as IFN antagonists, using chimeric HMPV expressing M2 proteins of either RSV or AMPV/C.

Chimeric HMPV expressing the M2 proteins of either RSV or AMPV/C were attenuated in IFN-competent cells but did not activate the IFN response and did not contain hypermutated genomes. This demonstrated that the M2 proteins of pneumoviruses have a similar function in the prevention of hypermutated genomes and activation of the IFN response. However, the attenuated replication of the chimeric viruses does indicate a role for these proteins in the replication process. The attenuation of both chimeric viruses indicated an interaction of M2 proteins with other (autologous) viral proteins for optimal replication. The higher level of attenuation of HMPV_RSV M2_ than HMPV_AMPV/C M2_ could be explained by the closer relationship between AMPV/C and HMPV than between RSV and HMPV.

To address the function of M2-2 proteins separately from M2-1, chimeric HMPVs were generated where M2-2 protein expression was expressed from the third position in the genome of HMPV with ablated M2-2 protein expression from the original position. Expression of HMPV M2-2 in the third position of the HMPV_StopM2-2_ genome restored the wild-type phenotype, indicating that M2-2 protein expression from a more proximal position is as efficient as expression from the original secondary ORF of the M2 gene. However, expression of the AMPV/C or RSV M2-2 protein from the third position of the genome did not restore the wild-type phenotype. These data demonstrate that expression of autologous M2-1 and M2-2 proteins is necessary to prevent the accumulation of mutations, as well as induction of the IFN response. The co-expression of both M2 proteins as a consequence of coupled translation of the M2 ORFs, as shown for RSV and AMPV (18, 19), does not appear to be required for the function of the M2 proteins, given that HMPV*_HMPV M2-2_ was phenotypically indistinguishable from HMPV_WT_.

Previously, it was shown that all HMPV mutants that contained hypermutated genomes also had an accumulation of DIs in the virus stocks (11). Although the presence of DIs in M2-2 chimeric viruses was not shown directly in the present study, it is likely that the chimeric viruses with hypermutated genomes also contained DIs, which subsequently could be responsible for activation of the IFN response. To study the effect of HMPV M2 when expressed from the RSV genome, HEp-2 cells were chosen because RSV replicates most efficiently in these cells. To compare the phenotypes of both HMPV and RSV chimeras, HEp-2 cells were used for all experiments. Previously, the induction of an IFN response by HMPV with ablated M2-2 protein expression was shown in A549 cells, and the observed levels of induced IFN production in HEp-2 cells were similar as those previously described. It is unlikely that the concerted function of the M2 proteins is different in other cell types, but this cannot be excluded.

The M2 proteins of pneumoviruses are known for their role in transcription and replication. Pneumoviruses have a transcription polarity from 3′ to 5′ ends, where gene-start (GS) and gene-end (GE) sequences, flanking each gene, are the transcription initiation and termination sites for each sequential ORF (20). As introducing gene boundaries at a non-natural location in the genome might affect replication kinetics or transcription profiles, we included a control virus where the HMPV M2-2 gene was placed under the control of the artificially introduced gene boundaries at the third position in the genome ofHMPV _stopM2-2_. Compared with the wild-type virus, no changes were observed in (i) replication kinetics, (ii) interferon induction, (iii) mutation rates, and (iv) transcription profiles. This indicates that the polymerase complex interacted with the introduced GS/GE signals in a similar way as in the wild-type virus. Therefore, the changes in phenotype observed for the other chimeras, using the same GS/GE signals at this position in the genome were not due to the introduction of the GS/GE signals at this non-natural location.

The M2-1 protein of RSV has been described as a transcription anti-terminator or elongation factor (21, 22), causing read-through at GE signals during transcription. A similar function has been proposed for the M2-1 protein of HMPV, but unlike the M2-1 protein of RSV, the M2-1 protein of HMPV is not essential for the transcriptional process, although its presence does improve transcription efficiency (23–25). The M2-2 protein of both HMPV and RSV has been described to regulate the switch between transcription and replication (16, 23, 26). Specifically, overexpression of the M2-2 protein of both pneumoviruses has been suggested to inhibit the viral transcription and replication (27, 28). Here, we show that the HMPV M2-2 protein affects the transcriptional profile. Replacement of the HMPV M2-2 protein by that of RSV or AMPV/C did not result in recovery of the wild-type phenotype, which could be explained by a low homology between the M2-2 proteins of the different pneumoviruses. Although the zinc-binding domains in M2-1, relevant for the role of M2-1 in particular, are conserved among pneumoviruses, the M2-2 proteins are highly variable between Pneumoviruses, 18%–19% between HMPV and RSV and 56% between HMPV and AMPV/C (29). Only when both the homologous M2-1 and M2-2 proteins of either AMPV/C or RSV were present, the transcription profile was restored. These results indicate a concerted action of autologous M2-1 and M2-2 proteins in the transcription process. The amino acid differences between the different M2 proteins of these pneumoviruses might indicate the reason why only in the presence of the autologous M2-1, the M2-2 protein can function adequately.

Previously, Groen *et al* showed that the genomes of M2-2 deletion mutants contained A-to-G and T-to-C mutations, demonstrated to be the result of ADAR1 editing, in the context of DIs (11). Here, we show that abolishing the transcription regulation function of the HMPV M2 proteins results in genome hypermutation, and thereby activation of the IFN response, likely due to the DIs. These DIs have been suggested to result from the errors made by the viral polymerase complex, as described for other RNA viruses (30). This editing pattern is similar to that observed for measles viruses (MeV) lacking the C protein (31, 32). In contrast, the genomes of Sendai virus (SeV) lacking C protein expression did not reveal A-to-G and T-to-C mutation patterns, although the virus stocks did contain DIs (33). Indeed, the C proteins of MeV, SeV, and parainfluenzavirus type 1 (PIV-1) have been suggested to control the polymerase processivity and replication (33–36). Additionally, these viral C proteins also function as IFN antagonists (reviewed in [37]). It remains unclear whether M2 proteins of pneumoviruses, besides having a transcriptional function, also function as bona fide IFN antagonists. However, it is unlikely since the M2-2 protein of HMPV could not replace the role of RSV NS1 and NS2 in the present study in RSV chimeric viruses lacking NS1 and NS2 proteins.

Because pneumovirus mRNAs are expressed as a polar transcriptional gradient (21, 38–40), deletion of any gene from the 5’ proximal site of the genome could increase expression levels of downstream genes. In this study, the attenuation of RSVΔNS1+2 could in part be due to disruption of the transcription profile, as suggested previously (41). The partial recovery of the wild-type replication phenotype by RSVΔNS1+2_AV NS1,_ RSVΔNS1+2_MPV N_, and RSVΔNS1+2_MPV M2-2_ could be due to partial restoration of the polar transcription gradient. However, the wild-type RSV genome contains two genes (and four GS and GE signals) at the 5’ proximal site, whereas the chimeras contained only one gene (and two GS and GE signals) at this position, which might explain the incomplete restoration. Although expression of the HMPV N and IAV NS1 proteins by the chimeric viruses was confirmed, an antibody that recognized the HMPV M2-2 protein was not available. In-house generated polyclonal guinea pig sera raised against HMPV, as well as peptide-based, affinity purified commercial antibodies against HMPV M2-2, failed to recognize the M2-2 protein, similar to a previous report (27). Therefore, instead of protein expression, HMPV M2-2 mRNA expression was confirmed by RT-PCR of the HMPV M2-2 gene. However, whether this mRNA is translated into protein could not be experimentally validated.

Expression of the M2-1 or other viral proteins by the M2-2 deletion mutant or chimeric viruses has not been evaluated. However, HMPV without M2-1 expression is non-viable, indicating that the virus must at least express the M2-1 protein. Whether the expression levels of M2-1 or other viral proteins differ for the chimeric viruses remains unclear, but it is likely that M2-1 expression is as dependent on M2-2 expression as the other viral genes.

The data were obtained using the recombinant NL/1/00 virus, the prototype virus of lineage A1. The recombinant virus was generated with the sequences of the original isolate and is therefore a representative of lineage A viruses. In addition, the M2-2 genes are highly conserved among HMPV genotypes, with 99% amino acid sequence homology between viruses within one lineage (A1, A2, B1, or B2), 96% to 98% between viruses within serotype A or B, and 89% to 91% between serotypes A and B. This indicates that the M2-2 protein of NL/1/00 is a representative of at least serotype A and most likely also serotype B.

In conclusion, this study shows that a concerted function of autologous pneumovirus M2-1 and M2-2 proteins during transcription prevents hypermutation of the genome, and possibly accumulation of DIs, thereby preventing the activation of IFN response. We could not provide definitive evidence that M2 proteins function as bona fide IFN antagonists. The primary role of M2 proteins appears to be the regulation of transcription and replication.

## MATERIALS AND METHODS

### Cloning and rescue of recombinant HMPV and RSV

For cloning of recombinant HMPV expressing the RSV or AMPV/C M2 ORFs at the original position in the HMPV genome, synthetic DNA fragments of position 4,460–7,438 from the HMPV NL/1/00 genome (AF371337.2) with the M2 gene exchanged for that of RSV (human respiratory syncytial virus strain A2, ATCC VR-1540) or AMPV/C (Avian pneumovirus strain Colorado, AY590688.1) were ordered from Baseclear, Leiden, the Netherlands. The synthetic DNA fragments were digested using StuI and BsrGI restriction sites and were subsequently used to clone the DNA fragment into the NL/1/00 genomic plasmid. For cloning of chimeric HMPV_StopM2-2_ viruses coding for the HMPV, RSV, or AMPV/C M2-2 proteins (either with or without a flag tag) in between the *P* and M genes, the gene of interest (GOI) was PCR amplified using primers with a 5’ tail sequence containing the BfuAI restriction site. The flag tag sequence was incorporated in the 5’ tail of the reverse primer for cloning GOIs with a flag tag sequence. The BfuAI restriction sites were then used to clone the fragment into a vector containing an NL/1/00 partial genome sequence of position 943–3,589 (from restriction sites ApaI to PmiI). This vector contained two BsmBI restriction sites flanked by the gene-start and gene-end sequences of the HMPV *P* gene located in between the *P* and M genes. This vector was then used to clone the GOI into the rHMPV NL/1/00 genomic plasmid using the ApaI and PmiI restriction sites, resulting in the viruses as shown in Figures 1A and 3A.

The pBr-322 vector containing the complete RSV A2 clone T306 genome was a kind gift from Professor Dr. A.G.P. Oomens (Department of Veterinary Pathology, Oklahoma State University). This plasmid was used as template DNA for PCR amplification of the RSV N, P, M2-1, and L genes, used to clone supporting pCITE plasmids encoding RSV N, P, M2-1, and L. Additionally, the pBr-322 plasmid was used as template DNA for PCR amplification of a ~ 5 kb fragment of the RSV genome, from the KpnI restriction site in the pBr-322 vector (just upstream of the leader sequence) to the XhoI restriction site at nucleotide position 4481, which was cloned into a pBluescript (pBSK) vector. This plasmid was used to generate RSVΔNS1+2 by circle PCR amplification using a reverse primer upstream of the NS1 gene and a forward primer downstream of the NS2 gene. These primers contained a 5’ tail with a BamHI site, which resulted in the following plasmid: pBSK plasmid–RSV Leader–gene start NS1–BsmHI–BamHI–BsmBI sites–gene end NS2–N–*P* (pBSK-partialRSV). To clone a GOI into this plasmid, a PCR product was generated using primers with a 5’ tail sequence containing a BsmBI site. The PCR product was subsequently cloned in the pBSK-partialRSV vector using the BsmBI sites, which resulted in RSVΔNS1+2 with an inserted GOI in the pBSK-partialRSV vector, for example, the Influenza A virus NS1 gene (Influenza A/Puerto Rico/8/1934 H1N1). This plasmid was then used to clone the fragment of the RSV genome containing the GOI into the pBr-322 plasmid containing the entire RSV genome, resulting in viruses as shown in Figure 6A.

HMPV rescue was performed as described previously (42), and RSV rescue was performed similarly to that of HMPV. Viruses were passaged only two times in Vero-118 cells at an MOI of 0.01 and purified by ultra-centrifugation as described previously (42).

### Cells and viruses

Vero-118, A549, and 293 T cells were cultured as described previously (42). All cells were kept at 37°C with 5% CO_2_ in a humidified incubator. Periodically, cells were tested and confirmed to be mycoplasma free. All media and supplements were purchased from Gibco (Life Technologies, Bleiswijk, The Netherlands). HEp-2 cells were cultured in Dulbecco’s modified Eagle’s medium (DMEM) containing 10% fetal calf serum (FCS), 100 IU penicillin/mL, and 100 µg streptomycin/mL. rHMPV NL/1/00 chimeric virus stocks were generated as described previously (42). RSV and HMPV titers were determined by end-point dilution in Vero-118 cells, expressed as TCID_50_/mL, calculated as described previously (43).

### Replication kinetics, RNA isolation, and quantitative PCR

Replication kinetics, RNA isolation, and quantitative PCR were performed as described previously (42, 44). The primers and probe used to detect the β-Actin gene are described by Spann *et al.* (45). IFN-β mRNA-fold induction in inoculated cells was compared with mock-inoculated cells and quantified using the ΔΔC_t_ method (46).

RNA levels of the HMPV N gene were detected using forward primer 5′-CATATAAGCATGCTA TATTAAAAGAGTCTC-3′, reverse primer 5′-CCTATTTCTGCAGCATATTTGTAATCAG-3′, and probe 5′-TGCAATGATGAGGGTGTCACTGCGGTTG-3′. RNA levels of the HMPV L gene were detected using forward primer 5′-TATTGGAGAAGGGGCAGGAAAT-3′, reverse primer 5′-ATTGAAAGCCCTTCACCGCTAT-3′, and probe 5′-CAGGATATTCACATGCTGTTCTGGCCATC-3′. The RNA expression levels of N and L were determined initially as C_T_ values by qRT-PCR, which were later correlated to TCID_50_ equivalents for N and L by using a standard curve derived from a virus stock of HMPV NL/1/00 titrated on Vero-118 cells (Fig. S2). A similar sensitivity of the N and L qRT-PCR assays allowed for the calculation of the N/L ratios shown in Figure 5.

### FACS analysis of infected cells

At time points as specified for each experiment, the cells were trypsinized and washed with 2% FCS in PBS. Immunostaining of HMPV-inoculated cells was performed as described previously (11). Cells inoculated with RSV were stained with Anti-Respiratory Syncytial Virus Antibody (2F7) (Santa Cruz, sc-101362) at a 1:250 dilution for 1 h followed by a 1:100 diluted polyclonal Rabbit-anti-mouse immunoglobulins/FITC-conjugated antibody (DAKO, F0313). The cells were fixed for 20 min in 2% paraformaldehyde in PBS, and the percentages of infected cells were quantified using a FACS Lyric machine (BD Biosciences).

### Western blot assay

Western blot assays were performed as described previously (11). Primary monoclonal mouse anti-influenza A NS1 antibody (sc-130568, Santa Cruz Biotechnology) was used to at a 1:1,000 dilution in PBS containing 1% milk and 0.1% Tween-20, and secondary polyclonal rabbit anti-mouse IgG-HRP was used as secondary antibody at a 1:1000 dilution.

### Validation of HMPV M2-2 mRNA expression by chimeric RSV

HEp-2 cells were inoculated at an MOI of 3 for HMPV and 0.5 for RSV. At 24 hpi, cells were lysed, and mRNA was isolated using the Roche mRNA isolation kit according to the manufacturer’s instructions. cDNA synthesis was performed using SuperScript IV Reverse Transcriptase (Invitrogen) according to the manufacturer’s protocol using an HMPV M2-2-specific reverse primer (5’-ctaacttaagtaagccttga-3’). The cDNA was then used for a HMPV M2-2-specific PCR using the PfuUltra II Fusion HS DNA polymerase (Agilent) according to the manufacturer’s instructions with forward primer 5’-atgactcttcatatgccttg-3’ and reverse primer 5’-gccttgacatatataatttc-3’. PCR products were run on a 1.5% agarose gel and imaged using a BioRad ChemiDoc MP imaging system.

### Immunofluorescence assay

A549 cells were inoculated at an MOI of 3 for HMPV and chimeric RSVs, and at an MOI of 0.5 for recombinant RSV_WT_. At the indicated time points, the cells were fixed for 15 min with 4% paraformaldehyde in PBS (Santa Cruz Biotechnology). The cells were permeabilized and blocked simultaneously for 30 min in PBS + 10% normal goat serum (NGS, ThermoFisher Scientific) + 0.2% triton-X100 (Sigma-Aldrich). The cells were stained with primary or secondary antibodies diluted in PBS + 10% NGS for 1 h at room temperature in the dark. The following dilutions of antibodies were used: anti-flag antibody (Sino Biological, 100233-MM01), polyclonal Rabbit-anti-mouse immunoglobulins/FITC conjugated antibody (DAKO, F0313) 1:100, polyclonal guinea pig sera anti-HMPV (in-house generated) 1:100, and Anti-Guinea Pig IgG-FITC (Sigma-Aldrich, F6261-1ML) 1:100. Nuclei were stained using Hoechst 33342 solution (20 mM) (ThermoFisher Scientific) at a 1:10.000 dilution for 3 min at room temperature.

### Illumina sequencing of virus genomes and data analysis

Library preparation and Illumina sequencing were performed as described previously (11, 47). Reads were aligned to the reference using CLC genomic workbench software (V 24.0.3). A custom Python (3.9, https://www.python.org/) script was used to iterate through reference-based alignments using Pysam (0.21, https://www.github.com/pysam-developers/pysam) and extract the count of all nucleotides from positions covered by at least 100 reads. For each position, the number of nucleotide substitution per 1,000 nucleotides from the reference to each of the three non-reference nucleotides were calculated. The rates across all positions were averaged and plotted using pandas (2.0, https://pandas.pydata.org/) and Plotly (5.15, https://plotly.com/python/) for Figures 2 and 4.

### Statistical analysis

Statistical analyses were performed using Graph Pad Prism 10.4.0 (GraphPad Software Inc., San Diego, CA, USA). For analysis of replication kinetics, the area under the curve (AUC) was determined and analyzed using one-way analysis of variance (ANOVA). For comparison of the induction of type I interferon, unpaired *t*-tests were used to determine significant differences. A *P*-value < 0.05 was considered to be significant.

## Data Availability

Data generated and used in this study are available upon request from the corresponding author.

## SUPPLEMENTAL MATERIAL

Supplemental figures jvi.01243-24-s0001.docx Figures S1 to S3.

ASM does not own the copyrights to Supplemental Material that may be linked to, or accessed through, an article. The authors have granted ASM a non-exclusive, world-wide license to publish the Supplemental Material files. Please contact the corresponding author directly for reuse.
