# Supplementary material for: The role of M2 proteins of pneumoviruses in transcription regulation, prevention of hypermutation, and activation of the type I interferon pathway
Source: J Virol. 2025 Jan 21;99(2):e01243-24. doi: 10.1128/jvi.01243-24 (PMC11852930; doi:10.1128/jvi.01243-24)
Supplement: Supplemental figures — Figures S1 to S3. [file jvi.01243-24-s0001.docx]

**
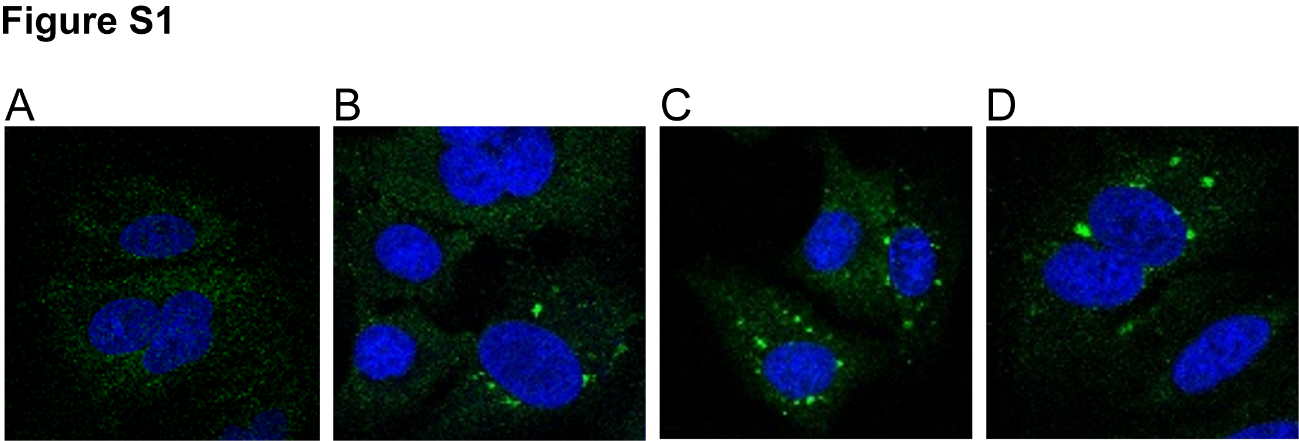
**

**
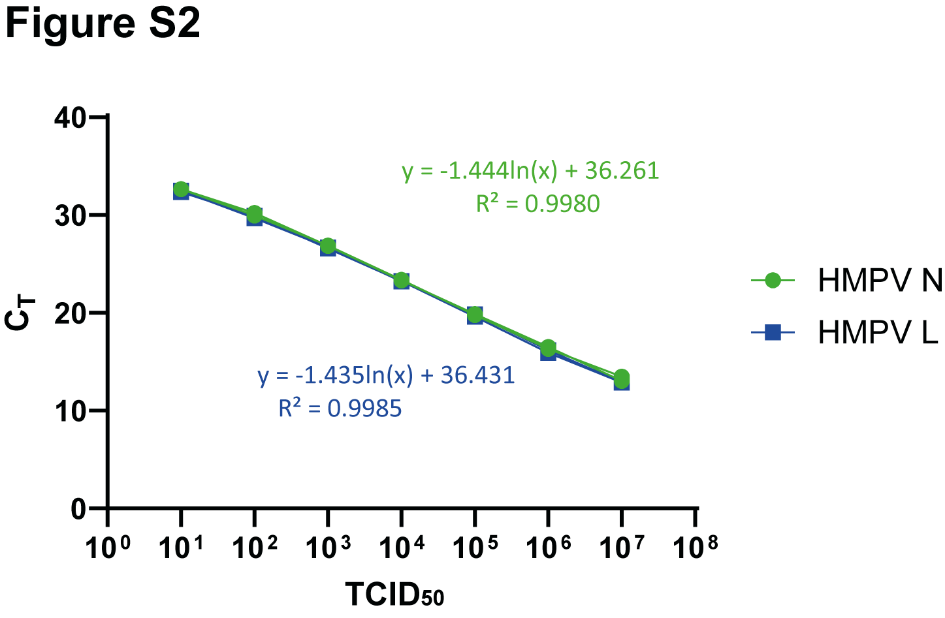
**

**
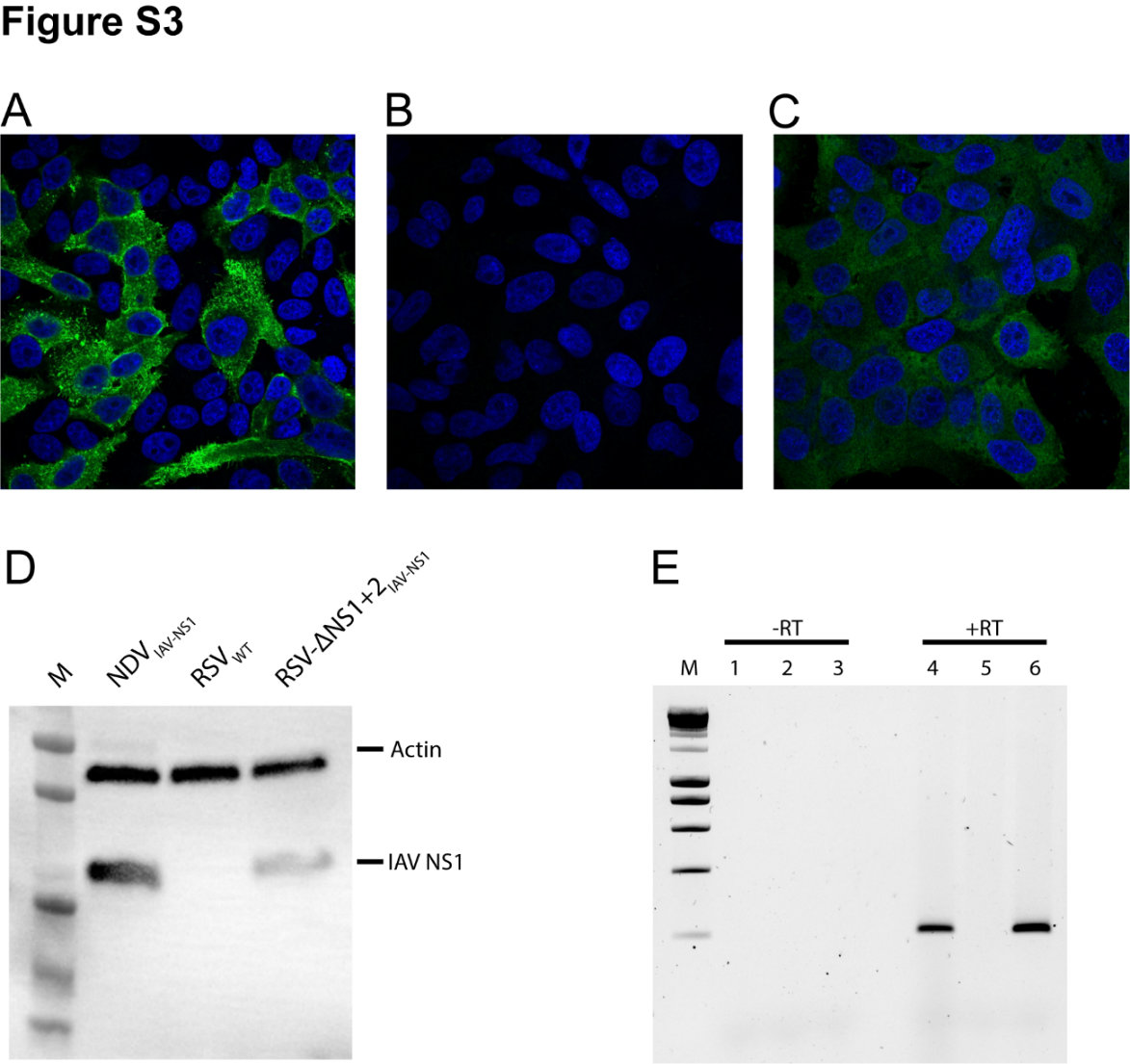
**

**Supplementary figure legends**

**Figure S1.** Confocal micrograph of A549 cells inoculated with HMPV_WT_ and chimeric HMPVs at 48 hpi at a 400x magnification. Cells inoculated with (A) HMPV_WT_, (B) HMPV*_HMPV M2-2-flag_, (C) HMPV*_RSV M2-2-flag_, or (D) HMPV*_AMPV/C M2-2-flag_ were stained with an anti-flag antibody (green) and Hoechst (blue).

**Figure S2.** Standard curves obtained from the serial TCID_50_ dilution of a virus stock of HMPV NL/1/00 titrated on Vero-118 cells, from which RNA was isolated and the C_T_ values were determined in duplo by qRT-PCR with two primer sets for the detection of N and L genes, respectively.

**Figure S3.** (A-C) Confocal micrograph of A549 cells inoculated with HMPV_WT,_ RSV_WT,_ and RSVΔNS1+2_HMPV N_ at 24 hours post inoculation at a 400x magnification. Cells inoculated with HMPV_WT_ (A), RSV_WT_ (B), or RSVΔNS1+2_HMPV-N_ (C) were stained with a polyclonal antibody against HMPV (green) and Hoechst (blue). (D) Western blot analysis of influenza A virus (IAV) NS1 protein expression by RSVΔNS1+2_IAV-NS1_ in A549 cells. Actin was used as a loading control and NDV_IAV-NS1_ was used as an expression control for IAV NS1 (46). (E) mRNA expression of the HMPV M2-2 gene expressed by RSVΔNS1+2_HMPV-M2-2._ mRNA was isolated from inoculated HEp-2 cells and subjected to cDNA synthesis, either with or without reverse transcriptase (-RT and +RT, respectively), followed by PCR of the HMPV M2-2 gene. Lanes on the gel represent: HMPV_WT_ (lane 1, 4), RSV_WT_ (lane 2, 5), RSVΔNS1+2_HMPV-M2-2_ (lane 3, 6).
